# Recommendations for Defining and Reporting Adherence Measured by Biometric Monitoring Technologies: Systematic Review

**DOI:** 10.2196/33537

**Published:** 2022-04-14

**Authors:** Iredia M Olaye, Mia P Belovsky, Lauren Bataille, Royce Cheng, Ali Ciger, Karen L Fortuna, Elena S Izmailova, Debbe McCall, Christopher J Miller, Willie Muehlhausen, Carrie A Northcott, Isaac R Rodriguez-Chavez, Abhishek Pratap, Benjamin Vandendriessche, Yaara Zisman-Ilani, Jessie P Bakker

**Affiliations:** 1 Department of Medicine Division of Clinical Epidemiology and Evaluative Sciences Research, Weill Cornell Medical College Cornell University New York, NY United States; 2 Sidney Kimmel Medical College at Thomas Jefferson University Philadelphia, PA United States; 3 Novartis Pharmaceuticals Corporation East Hanover, NJ United States; 4 Health Platforms, Verily Life Sciences Cambridge, MA United States; 5 Pfizer Berlin Germany; 6 Giesel School of Medicine at Dartmouth College Hanover, NH United States; 7 Koneksa Health New York, NY United States; 8 AstraZeneca Pharmaceuticals LP Gaithersburg, MD United States; 9 SAFIRA Clinical Research Cloughjordan Ireland; 10 Pfizer Inc Cambridge, MA United States; 11 ICON plc Blue Bell, PA United States; 12 CAMH Krembil Center for Neuroinformatics Toronto, ON Canada; 13 Vector Institute Toronto, ON Canada; 14 Biomedical Informatics and Medical Education University of Washington Seattle, WA United States; 15 Institute of Psychiatry, Psychology, and Neuroscience, Kings College London London United Kingdom; 16 Byteflies Antwerp Belgium; 17 Department of Electrical, Computer, and Systems Engineering, Case Western Reserve University Cleveland, OH United States; 18 Department of Social and Behavioral Sciences; College of Public Health, Temple University Philadelphia, PA United States; 19 Signifier Medical Technologies Needham, MA United States

**Keywords:** digital medicine, digital measures, adherence, compliance, mobile phone

## Abstract

**Background:**

Suboptimal adherence to data collection procedures or a study intervention is often the cause of a failed clinical trial. Data from connected sensors, including wearables, referred to here as biometric monitoring technologies (BioMeTs), are capable of capturing adherence to both digital therapeutics and digital data collection procedures, thereby providing the opportunity to identify the determinants of adherence and thereafter, methods to maximize adherence.

**Objective:**

We aim to describe the methods and definitions by which adherence has been captured and reported using BioMeTs in recent years. Identifying key gaps allowed us to make recommendations regarding minimum reporting requirements and consistency of definitions for BioMeT-based adherence data.

**Methods:**

We conducted a systematic review of studies published between 2014 and 2019, which deployed a BioMeT outside the clinical or laboratory setting for which a quantitative, nonsurrogate, sensor-based measurement of adherence was reported. After systematically screening the manuscripts for eligibility, we extracted details regarding study design, participants, the BioMeT or BioMeTs used, and the definition and units of adherence. The primary definitions of adherence were categorized as a continuous variable based on duration (highest resolution), a continuous variable based on the number of measurements completed, or a categorical variable (lowest resolution).

**Results:**

Our PubMed search terms identified 940 manuscripts; 100 (10.6%) met our eligibility criteria and contained descriptions of 110 BioMeTs. During literature screening, we found that 30% (53/177) of the studies that used a BioMeT outside of the clinical or laboratory setting failed to report a sensor-based, nonsurrogate, quantitative measurement of adherence. We identified 37 unique definitions of adherence reported for the 110 BioMeTs and observed that uniformity of adherence definitions was associated with the resolution of the data reported. When adherence was reported as a continuous time-based variable, the same definition of adherence was adopted for 92% (46/50) of the tools. However, when adherence data were simplified to a categorical variable, we observed 25 unique definitions of adherence reported for 37 tools.

**Conclusions:**

We recommend that quantitative, nonsurrogate, sensor-based adherence data be reported for all BioMeTs when feasible; a clear description of the sensor or sensors used to capture adherence data, the algorithm or algorithms that convert sample-level measurements to a metric of adherence, and the analytic validation data demonstrating that BioMeT-generated adherence is an accurate and reliable measurement of actual use be provided when available; and primary adherence data be reported as a continuous variable followed by categorical definitions if needed, and that the categories adopted are supported by clinical validation data and/or consistent with previous reports.

## Introduction

### Background

Suboptimal adherence to clinical study procedures and/or a study intervention is often the root cause of a failed clinical trial, as it contributes to missing data; dilutes the effect of the intervention, thereby reducing statistical power; and may contribute to selection bias [1-3]. Compounding this issue is the fact that adherence is challenging to measure and account for during the analysis [4]. For example, self-reported medication adherence is straightforward to capture but is generally not a reliable measure of actual use; pill counts and pharmacy refill rates are imperfect surrogate measurements, and physical tests such as blood or urine biomarkers are expensive and difficult to administer throughout a trial [5-7]. Connected sensor technologies, such as mobile health and wearables, represent a potential solution for measuring adherence to a therapeutic intervention accurately, given that they are capable of collecting continuous, sensor-based data in real-world settings. Such technology has been increasingly used to capture trial end points [8,9]; therefore, in addition to monitoring adherence to an intervention, there exists an opportunity to monitor adherence to data collection procedures.

When conducting a clinical trial, the goal is to maximize adherence to both study procedures and interventions. However, efforts focused on increasing adherence cannot be developed until the determinants of adherence are understood. In turn, the reasons for suboptimal adherence cannot be identified unless adherence is adequately measured and reported in studies that use biometric monitoring technologies (BioMeTs), defined previously as connected digital tools that process data captured by mobile sensors using algorithms to generate measures of behavioral or physiological function [10]. A growing body of literature has offered standards and guidance to improve digital medicine study design and reporting quality [8-15]; however, best practices regarding measurement and reporting of BioMeT adherence—the extent to which the tool itself or an associated intervention is used as designed—are not as clearly conceptualized [4,16].

### Objectives

A research working group from the Digital Medicine Society was formed to conduct a systematic literature review of published studies reporting adherence captured by BioMeTs to (1) identify studies that have used these tools to capture adherence to data collection procedures and/or study interventions, (2) describe the various methods used to measure adherence, and (3) compare the definitions of adherence reported in the literature. We view this description of the current state of the art as a critical first step toward identifying the determinants of adherence, in order to develop adjunct interventions to maximize adherence, ultimately contributing to improving the efficiency of clinical trials using novel technology.

## Methods

### Literature Search

The PubMed search terms were designed in five layers as follows: (1) used a BioMeT (layer A), (2) reported adherence or compliance (layer B), (3) were clinical studies (layer C), (4) reported original data (layer D), and (5) were published between January 1, 2014, and November 19, 2019 (layer E). Layers B, C, D, and E were based on indexing data available in PubMed, such as Medical Subject Headings terms and publication types. Layer A was designed to identify studies using a BioMeT and comprised 3 Medical Subject Headings terms as well as 34 keywords including *tracker*, *implantable*, *watch*, *mobile*, and *sensor* (see Multimedia Appendix 1 for complete search terms). When developing Layer A, our goal was to be sensitive rather than specific, as we anticipated variability in how BioMeTs are described in the literature.

We have adopted the term *adherence* rather than *compliance* or *concordance* throughout this manuscript, although we recognize that these terms cover a range of inconsistent definitions including patient-driven decisions and behaviors, passively conforming to medical advice, and the extent to which a research participant follows a study protocol [17]. Specifically, we included BioMeTs that measured the use of the tool itself, such as a wrist-worn device containing a skin capacitance sensor to monitor the duration of use, in addition to BioMeTs that measured the use of a diagnostic or therapeutic tool, such as a temperature sensor to measure the use of a dental appliance.

### Systematic Review

We developed a Population, Intervention, Comparison, Outcomes, and Study design (PICOS) framework [18] to formulate the eligibility criteria for prospective studies of human participants (Textbox 1). Each study deployed at least one BioMeT outside the clinical setting or a testing facility, for which a quantitative, nonsurrogate, and sensor-based measurement of adherence was reported.

**Eligibility criteria adopted for literature screening in Population, Intervention, Comparison, Outcomes, and Study design order.**

**Eligibility criteria**

**Population**

- Identify human studies
- Identify studies capturing in vivo data

**Intervention**

- Identify studies that used at least one biometric monitoring technology (BioMeT):
  The tool must be used for purposes of measurement, diagnosis, and/or treatment of a behavioral or physiological function related to a disease state or physiological condition.
  The tool must be mobile, meaning that it is capable of collecting data in real-world settings without oversight from trained personnel or staff.
  The tool must be connected, meaning that there is a method to move data from the tool to the clinical or laboratory for analysis.
  The tool must capture data via sensors of a physical property.
- Identify studies that captured BioMeT data outside of the clinical or laboratory setting.

**Comparison**

- Not applicable

**Outcomes**

- Identify studies that reported adherence:
  The tool must measure adherence directly, rather than surrogate data associated with adherence.
  The adherence data must be quantitative.
  The adherence data must be sensor-based rather than based on self-report, observation, and/or based on manual adjustment or scoring.

**Study design**

- Identify studies reporting primary analyses of prospective data collection

Within the aforementioned definition of a BioMeT [10], the term *connected* was interpreted to include any wired or wireless transfer of data; thus, products that used a physical connection for data transfer were included, but devices that only displayed data on a user interface were excluded. Similarly, we interpreted the term *mobile* broadly and included wearables, proximal sensors, ingestibles, implantables, and tools that require a brief interaction such as a smartphone. When considering *sensors*, we included only those that measured physical properties such as temperature, pressure, sound, or acceleration. Therefore, we excluded tools that contained a chronometer that relied on being turned on or off or analyses based on self-report or other subjective assessments.

We stipulated that the measurement of adherence must be quantitative, nonsurrogate, and sensor-based. We defined *nonsurrogate* as an unequivocal reflection of product use. For example, technologies such as smart pill bottles, in which a sensor records the time at which the lid is removed, were considered a surrogate measure of adherence because they do not measure the ingestion of the pills. In contrast, smart pills that combine a pharmaceutical agent with a digital radio-frequency emitter activated by chloride ions in the digestive system were considered as nonsurrogate adherence measurements, and therefore in scope. Finally, BioMeTs were excluded if the adherence data were based on self-report or observation or if any component of the adherence data required manual adjustment or scoring.

In total, 5 independent investigators (JPB, AC, DM, WM, and IMO) applied the PICOS criteria to a subset of 42 manuscripts for training purposes. The screening results were compared, discrepancies were discussed as a group, and the PICOS criteria were refined and clarified to optimize standardization during the remaining literature screening process. The remaining 898 manuscripts were then divided across 5 trained investigators for screening (LB, AC, WM, IMO, and BV), whereas a sixth investigator (JPB) independently screened a subset of 20% (180/898) of the manuscripts for quality assessment, as described below.

### Data Extraction and Analysis

Data extraction fields included the study aim, study design (observational or interventional), therapeutic area, country of data collection, participant demographics (age, sex or gender, and race or ethnicity), information related to the BioMeT (concept of interest as described previously [19], technology type, sensor or sensors, device make and model, software name and version), and adherence data (end point definition and units). End point definitions were identified as the primary metric by which the sample-level data were analyzed to describe BioMeT adherence; for example, the duration of use, the percentage of tasks completed, or the percentage of study participants achieving a specified use goal. Therapeutic areas were categorized according to the Clinical Data Interchange Standards Consortium list [20], with additional categories for healthy and overweight or obesity. The sample size extracted from each manuscript was either the number of participants contributing to the adherence data or, if not reported, the total sample size for the study.

We categorized the primary definition of adherence for each BioMeT according to decreasing levels of data resolution as follows: (1) duration of use, either as a unit of time or percentage (continuous variable); (2) the number of measurements completed or number of days containing a measurement (continuous variable); or (3) the percentage of study participants who achieved a use goal (binary variable). Each BioMeT was categorized as passive (tools designed for continuous use) or active (tools that require user engagement at defined time points). Active BioMeTs were further categorized as session-based tools, such as connected exercise equipment, or task-based tools, such as a smart scale. This distinction was made because duration-based adherence data cannot be extracted from tools that measure one-off tasks; therefore, the highest-resolution adherence data available from these tools are the number of tasks or measurements completed.

All data were presented with descriptive statistics.

## Results

### Literature Screening Results

**Figure 1:**
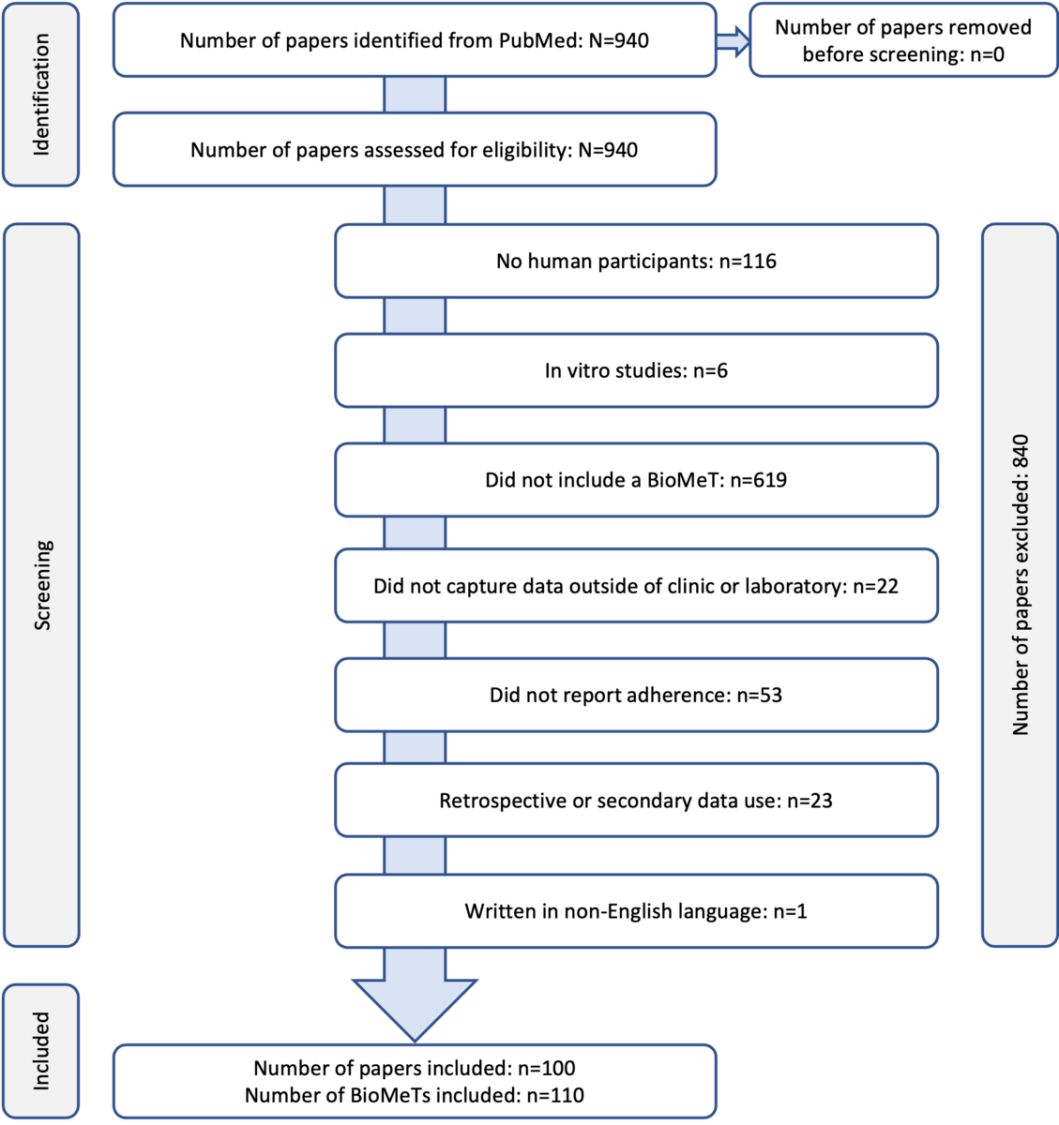
Literature screening results per PRISMA (Preferred Reporting Items for Systematic Reviews and Meta-Analyses) guidelines. Note that all papers were assessed for eligibility based on information contained in the abstract or full text. Papers were not screened based on the title only, as it was anticipated that many studies would include biometric monitoring technology (BioMeT) data as an exploratory end point and therefore, they would be not captured in the title.

The PubMed search identified 940 manuscripts, of which 100 (10.6%) were deemed eligible for inclusion in the systematic review after meeting the PICOS criteria (Figure 1; see Multimedia Appendix 2 for all 100 papers listed). Data were extracted from these 100 manuscripts as described earlier.

**Figure 2:**
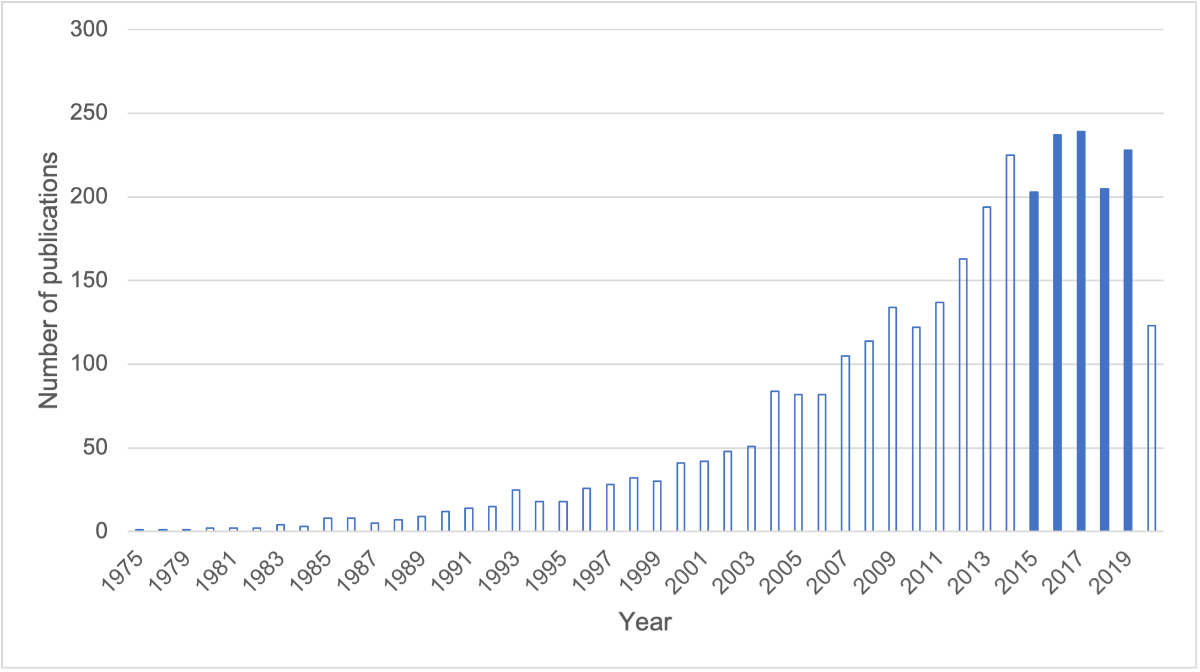
Number of publications captured by our literature search terms over time. The solid bars indicate the publications screened for inclusion in our systematic review.

After removing the date constraints from the PubMed search terms, we repeated the search by year to assess the number of publications captured from 1975 to 2020 (Figure 2).

### Literature Screening Quality Assessment

The large number of manuscripts identified during the PubMed search (N=940) precluded the ability of more than one independent investigator to review each against our PICOS criteria. As described earlier, of the 898 manuscripts remaining after assessment of the subset of 42 manuscripts identified for training, we randomly identified 180 (20%) manuscripts to be rescreened by an independent investigator (JPB) for quality assessment. During this process, of the 180 manuscripts, there were 13 (7%) instances in which there was disagreement regarding the classification of the manuscripts. Most of these disagreements resulted from ambiguity of reporting with respect to whether the tool in question met the definition of a BioMeT (8/13, 62% of the manuscripts), whether the device measured adherence via a sensor (1/13, 8% of the manuscripts), or the setting of data collection (1/13, 8% of the manuscripts). Finally, 23% (3/13) of the disagreements were errors in which the manuscript was inadvertently marked as not including human participants or based on in vitro analyses. An additional 8 (1.11%) manuscripts that were not part of the audit subset were marked by the reviewer as ambiguous; all of these were cross-checked by another investigator to determine eligibility.

### Descriptive Data

Table 1 summarizes the study design, sample size, and participant demographics of the 100 eligible studies. The sample size ranged from 10 to 128,037 participants, with an overall median of 60 participants (IQR 35-137). Most studies (92/100, 92%) used a single BioMeT; however, a second and third BioMeT was used in 6 (6%) and 2 (2%) manuscripts, respectively. Thus, 100 studies contributed data on 110 BioMeTs.

The manufacturer and/or model were reported for 90.0% (99/110) of the BioMeTs; however, only 30.9% (34/110) reported the software name and/or version. BioMeTs were categorized according to their concept of interest, with exercise or sleep (47/110, 42.7%) and sleep-disordered breathing (25/110, 22.7%) being the most common. These 2 categories also contained the highest number of BioMeT tool types; for example, exercise or sleep was captured by wearables, chest straps, smart clothing or footwear, and smartphones, whereas the sleep-disordered breathing category included positive airway pressure devices, chest straps for the treatment of positional obstructive sleep apnea, oral appliances, and implantable nerve stimulator devices. Notably, proximal sensors were considered in the scope, but none were captured in our systematic review.

**Table 1:** Study details, demographic data, and biometric monitoring technologies (BioMeTs) by therapeutic area.

					Therapeutic area of focus																	
					All (N=100)		Healthy (n=11)		Cardiovascular (n=17)			Endocrine (n=13)		Neural (n=10)		Overweight or obesity (n=6)		Respiratory (n=29)		Pain treatments (n=5)		Other^a^ (n=9)
**Study design, n (%)**																						
	Observational studies				26 (26)		3 (27)		1 (6)			4 (31)		3 (30)		1 (17)		9 (31)		1 (20)		4 (44)
	Interventional studies				74 (74)		8 (73)		16 (94)			9 (69)		7 (70)		5 (83)		20 (69)		4 (80)		5 (56)
Sample size (participants), median; range					60; 10-128,037		179; 42-1381		84; 40-1732			46; 10-234		22; 10-780		86; 11-174		70; 10-128,037		35; 10-68		56; 20-281
**Participant characteristics, n (%)**																						
	**Sex or gender**																					
			Females or women only^b^	9 (9)		3 (27)				1 (6)	0 (0)		0 (0)		3 (50)		0 (0)		0 (0)		2 (22)	
			Both sexes or genders	84 (84)		7 (64)				16 (94)	12 (92)		8 (80)		3 (50)		26 (90)		5 (100)		7 (78)	
			Not reported	7 (7)		1 (9)				0 (0)	1 (8)		2 (20)		0 (0)		3 (10)		0 (0)		0 (0)	
	**Age (years), n (%)**																					
		≥60		24 (24)		4 (36)			4 (24)		5 (38)		2 (20)		0 (0)		5 (17)		1 (20)		3 (33)	
		>21 to <60		57 (57)		5 (45)			9 (53)		4 (31)		5 (50)		3 (50)		22 (76)^c^		3 (60)		6 (67)	
		≤21		19 (19)		2 (18)			4 (24)		4 (31)^c^		3 (30)		3 (50)		2 (7)		1 (20)		0 (0)	
	**Race or ethnicity, n (%)**																					
		Reported		39 (39)		7 (64)			11 (65)		6(46)		2 (20)		4 (67)		3 (10)		2 (40)		4 (44)	
		Not reported		61 (61)		4 (36)			6 (35)		7 (54)		8 (80)		2 (33)		26 (90)		3 (60)		5 (56)	
**BioMeT tool type, n, (%)**																						
	Wearable				46 (42)		10 (91)		9 (41)			4 (27)		6 (60)		5 (83)		2 (7)		5 (83)		5 (50)
	Positive airway pressure device				18 (16)		—^d^		—			—		—		—		18 (60)		—		—
	Smart clothing				8 (7)		—		3 (14)			3 (20)		1 (10)		—		—		—		1 (10)
	Blood pressure monitor				6 (5)		—		5 (23)			1 (7)		—		—		—		—		—
	Chest strap				5 (5)		—		2 (9)			—		—		—		3 (10)		—		—
	Smartphone				5 (5)		—		1 (5)			—		2 (20)		1 (17)		—		—		1 (10)
	Oral appliance				5 (5)		—		—			—		—		—		2; 7%		—		3 (30)
	Glucometer; continuous				3 (3)		—		—			3 (20)		—		—		—		—		—
	Glucometer; noncontinuous				3 (3)		—		—			3 (20)		—		—		—		—		—
	Ingestible				2 (2)		—		1 (5)			—		—		—		—		1 (17)		—
	Implantable				2 (2)		—		—			—		—		—		2 (7)		—		—
	Smart scale				2 (2)		—		1 (5)			1 (7)		—		—		—		—		—
	Adhesive patch				1 (1)		1 (9)		—			—		—		—		—		—		—
	Exercise equipment				1 (1)		—		—			—		—		—		1 (3)		—		—
	Muscle trainer				1 (1)		—		—			—		—		—		1 (3)		—		—
	Hearing aid				1 (1)		—		—			—		1 (10)		—		—		—		—
	Home oxygen				1 (1)		—		—			—		—		—		1 (3)		—		—
	Total				110		11		22			15		10		6		30		6		10

^a^Other category included oncology, gastrointestinal, bone structure, anatomy, or orthodontics, pregnancy, and vocal cord dysfunction.

^b^No studies included only males or men.

^c^Each of these categories contained 1 study that reported age only qualitatively or by providing a range; all other studies reported an average age.

^d^No studies falling into that category.

### Adherence Data

Overall, we identified 37 unique definitions for the 110 BioMeTs. The most commonly reported definition (duration of use) was reported for 41.8% (46/110) of the tools; however, the next most common definitions (number or percentage of tasks completed and number or percentage of days with data) were reported for only 8.2% (9/110) and 6.4% (7/110) of the BioMeTs, respectively.

As shown in Table 2, each BioMeT was categorized as passive (69/110, 62.7%), session-based (24/110, 21.8%), or task-based (17/110, 15.5%). The duration of use was reported for 46% (32/69) of the passive BioMeTs and 75% (18/24) of the session-based BioMeTs. Of the task-based BioMeTs for which the duration of use could not be meaningfully reported, the highest resolution of adherence data (the number of measurements or days) was reported for 41% (7/17) of the BioMeTs. The lowest resolution of adherence data (achievement of a goal as a binary or categorical variable) was reported for 33.6% (37/110) of all BioMeTs.

**Table 2:** Adherence data resolution and definition captured by passive and active biometric monitoring technologies (BioMeTs).

Parameters			Highest resolution adherence data										Lowest resolution adherence data		
			Duration of use (based on a continuous variable)					Number of measurements or days used (based on a continuous variable)					Achievement of a goal (based on a binary variable)		
			Number of BioMeTs, n (%)		Number of unique adherence definitions		Number of BioMeTs, n (%)			Number of unique adherence definitions		Number of BioMeTs, n (%)			Number of unique adherence definitions
**Monitoring type**															
	Passive	32 (64)		4		16 (70)			7		21 (57)			19	
	Active; session-based	18 (36)		1		0 (0)			0		6 (16)			2	
	Active; task-based	N/A^a^		N/A		7 (30)			4		10 (27)			5	
All BioMeTs			50 (100)		4^b^		23 (100)			8^b^		37 (100)			25^b^
All BioMeTs apart from sleep-disordered breathing			30 (60)		4		23 (100)			8		32 (86)			24

^a^N/A: not applicable.

^b^These data are not simply the sum of the rows above, as there were instances where the same adherence definition was adopted for different tool types.

**Figure 3:**
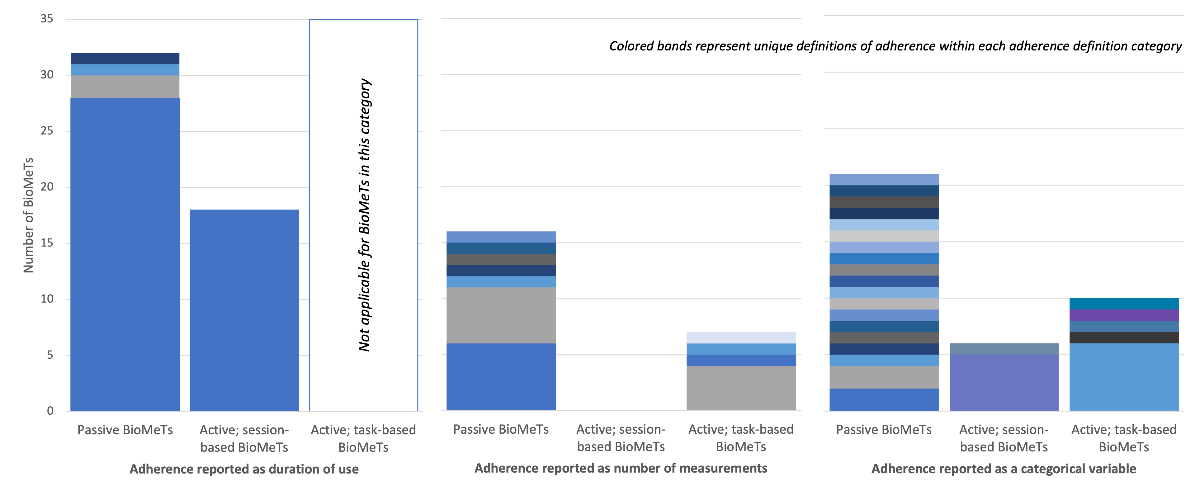
Uniformity of adherence definitions according to whether the biometric monitoring technology (BioMeT) was a passive, session-based, or task-based tool. Passive BioMeTs are those designed for continuous use. Active BioMeTs are those that require user engagement at defined time points, further categorized as session-based (for which duration of use is meaningful) versus task-based (for which the duration of use is not meaningful). The colored bands represent unique definitions of adherence within each bar. The colors are comparable across the bars within each category of adherence definition (duration of use, number of measurements, and categorical variables).

As shown in Figure 3, the number of unique definitions of adherence increased as the resolution of the reported data decreased. For example, adherence was reported as the duration of use (highest resolution) for 50 BioMeTs, 46 (92%) of which reported an actual unit of time, along with 3 other unique definitions of adherence, such as the duration of use in which a heart rate goal was achieved. In contrast, among the 37 BioMeTs for which adherence was reported as a categorical variable (lowest resolution), 25 unique definitions of adherence were identified. Sleep-disordered breathing was the BioMeT category with the most consistent definitions of adherence (2 definitions reported for 25 BioMeTs); however, the pattern of decreasing uniformity score alongside decreasing data resolution persisted after removal of these BioMeTs (Table 2).

## Discussion

### Principal Findings

The purpose of this review was to describe the various approaches taken to evaluate adherence to study procedures and/or interventions using BioMeTs in recent clinical studies and discuss best practices that can improve the reliability and comparability of adherence measurements to support further BioMeT evaluation and decision-making in both research and clinical care settings. Notably, we found that 29.9% (53/177) of the studies that used a BioMeT outside the clinical or laboratory setting failed to report a sensor-based, nonsurrogate, quantitative measurement of adherence, thus impeding a complete understanding of the study data. Among the 100 studies that reported sensor-based adherence data, we found substantial variability in terms of the definitions of adherence adopted, and that the degree of variability was associated with the resolution of the data reported. For example, when adherence was reported as a continuous time variable, the same definition of adherence was adopted for 92% (46/50) of the tools. However, when the adherence data were simplified to a categorical variable, we observed 25 unique definitions of adherence reported for 37 tools, and the most common definition was adopted for only 19% (7/37) of the BioMeTs. Examples of adherence definitions that were reported only once each within our data set include the percentage of participants with use <85% of the total time (passive BioMeT), the percentage of participants with use of ≥4 hours on ≥70 days (active, session-based BioMeT), and the percentage of participants completing readings on 100% of days (active, task-based BioMeT). All 3 of these adherence definitions were relevant and useful for the study in question; however, by adopting a specific threshold and reporting adherence as the percentage of the sample that achieved the goal, the adherence data were not readily interpretable against other studies. If adherence data were provided as higher-resolution variables, such as duration of use or number of readings, readers would be better positioned to make comparisons. Considering that adherence to any given procedure or intervention is a critical driver of desired behavior change and improved health outcomes in research and real-world settings [21], greater consistency in defining adherence may help more clearly associate BioMeT adherence to study outcomes and may offer a critical lens in the design and implementation of customized BioMeTs that are fit-for-purpose within their context of use.

In addition to consistent reporting of adherence, it is critical to understand exactly what digital medicine tools are used in a given study, consistent with the 2021 EVIDENCE (Evaluating Connected Sensor Technologies) Publication Checklist [11]. A complete description of the tool used ensures reproducibility, allows for meaningful comparisons across studies, and opens up the possibility of merging data across cohorts. Although the manufacturer or model (or both) were reported for 90.0% (99/110) of the tools captured in our review, this still leaves 10.0% (11/110) for which the tools were described in generic terms. Moreover, we found that the software name or version (or both) used for data processing was reported for only 30.9% (34/110) of the BioMeTs, indicating that the data cannot be reproduced even if the hardware details are known. We also noted key gaps in the descriptive data; most notably, only 41% (41/100) reported the ethnicity or race of participants, which is a persistent problem in clinical research [22,23]. Even among the 46 studies performed in the United States, for which there are clear guidelines for collecting and reporting race and ethnicity [24], 30% (14/46) of the studies did not provide these data. Age, sex or gender, and race or ethnicity are essential for understanding the representativeness of study samples and generalizability of findings and reflect only a subset of a broader set of characteristics such as socioeconomic information that must be captured and reported to understand how BioMeT adherence relates to issues of access, uptake, equity, and equality [25,26].

Differences across studies using BioMeTs are inevitable; however, ideally, there should be standardization and harmonization of collecting and reporting adherence to allow for the evaluation, interpretation, and statistical comparison of outcomes. Thus, although this study was not designed to develop standards, the aforementioned gaps and shortcomings have led us to recommend minimum reporting requirements and that consistent definitions or units for adherence should be adopted. Specifically, we recommend that (1) quantitative, nonsurrogate, sensor-based adherence data be reported for all BioMeTs when feasible; (2) a clear description of the sensor or sensors used to capture adherence data, the algorithm or algorithms that convert sample-level measurements to a metric of adherence, and the analytic validation data demonstrating that BioMeT-generated adherence is an accurate and reliable measurement of actual use be provided when available; and (3) primary adherence data be reported as a continuous variable followed by categorical definitions if needed, and that the categories adopted are supported by clinical validation data or consistent with previous reports (or both). These recommendations are in addition to the minimum requirements recommended elsewhere, such as providing a description of verification, validation, and usability data explaining the fit-for-purpose characteristics of the BioMeT technology used within a specific context as well as detailed demographic and descriptive data for the study sample [10,11,15]. More detailed descriptions of our recommendations for reporting BioMeT adherence are provided in Table 3, which includes a case study that we identified as an exemplar that followed all included recommendations [27].

On a positive note, it is clear that BioMeTs have been increasingly deployed in clinical research studies. Repeating our PubMed search over successive years revealed that the number of publications captured in our search terms increased steadily between 1975 and 2005 and became increasingly prevalent until 2015. The reduced number of papers captured in 2020 may reflect a delay in PubMed indexing and possibly a reduced submission and/or acceptance rate during the initial stages of the COVID-19 pandemic. It is encouraging to observe adherence data reported from a wide range of monitoring, diagnostic, and therapeutic tools from studies conducted in 22 different countries. Furthermore, although accelerometry-based tools for estimating sleep and activity have been in use for several decades [28], our literature search captured adherence data for more recently developed tools, such as automated speech assessments [29] and upper-limb training systems for motor disorders [30].

**Table 3:** Recommendations for capturing and reporting adherence measured by biometric monitoring technologies (BioMeTs).

Identified gaps and recommendations			Case study [27]
**Gap 1: Quantitative, nonsurrogate, sensor-based adherence data were not reported in 29.9% of screened manuscripts that captured BioMeT data outside the clinical or laboratory setting.**			
	Recommendation 1: Investigators are encouraged to develop and/or use BioMeT sensors to capture sensor-based adherence data in addition to their primary purpose.	This study aimed to evaluate adherence to a physical activity among students recruited from 20 schools. Quantitative adherence data were derived from wrist-worn accelerometers, considered a direct reflection of wear-time.	
	Recommendation 2: Where feasible, we encourage investigators to collect and report adherence data that are a direct reflection of actual use, rather than a surrogate.	N/A^a^	
**Gap 2: BioMeT manufacturer or model and software information was missing for 10% and 68% of included tools, respectively.**			
	Recommendation 3: In addition to reporting the BioMeT manufacturer or model and software used for generating adherence data (where applicable), we recommend that investigators provide a clear description of the sensor or sensors capturing adherence data.	BioMeT model: GENEActiv wrist-worn device (ActivInsights Ltd). Sensor description: 3-axis accelerometer. Software: GENEActiv PC software (version 2.9), with subsequent signal processing performed in R-package (GGIR; version 1.2-2).	
	Recommendation 4: We recommend that investigators describe the algorithm or algorithms that convert sample-level measurements into a measurement of adherence. If a description is not available from the manufacturer, this should be stated.	The paper included the data sampling frequency (100 Hz); a description of the signal processing steps including calibration; the epoch length (5 seconds) over which the sample-level data were averaged; and the units (milligravitational units; m g). A description of the nonwear detection algorithm was summarized as, “Non-wear is estimated on the basis of the SD and value range of each axis, calculated for 60-min windows with 15-min sliding window. The window is classified as non-wear if, for at least two of the three axes, the SD is less than 13 mg or the value range is less than 50 mg.”	
	Recommendation 5: We recommend that investigators describe the analytic validation data supporting the adherence algorithm; that is, the data indicating that adherence per the BioMeT is an accurate estimate of actual use. If analytic validation data is not available, this should be stated.	A reference to previous verification and analytic validation work was included.	
**Gap 3: Heterogeneity of adherence definitions increased alongside decreasing resolution of adherence data reported.**			
	Recommendation 6: We recommend that investigators using BioMeTs that are either passive (designed to capture data passively over long periods) or session-based (designed for user engagement at certain time points, for which the duration of use is meaningful) report primary adherence as a continuous variable of time; that is, total minutes or hours or days, or average hours per day, days per week, and so on. Example of a passive BioMeT: smart clothing. Example of a session-based BioMeT: connected exercise equipment.	The BioMeT was categorized as passive, as the wrist-worn accelerometer was designed to capture data continuously over 3 separate periods of 7 days. Adherence was reported as the total hours of wear-time, and hours per day of wear-time.	
	Recommendation 7: We recommend that investigators using BioMeTs that are task based (designed for user engagement at certain time points, for which the duration of use is not meaningful) report primary adherence as a continuous variable; that is, the number of tasks or days completed. Example of a task-based BioMeT: connected scale.	N/A, as the BioMeT was categorized as passive rather than task based.	
	Recommendation 8: We recommend that categorical adherence data are reported only in addition to continuous adherence data; for example, the percentage of participants with use >x hours per day or percentage of participants completing >y tasks.	Categorical adherence data included the number of participants with ≥16 hours of wear-time per day.	
	Recommendation 9: We recommend that categorical definitions of adherence be based on clinical validation data indicating the level of adherence associated with a clinically meaningful change in the outcome of interest, when available. If clinical validation data are not available, this should be stated.	The investigators include a reference to previous work that adopted the threshold of ≥16 hours of wear-time per day and describe another study that compared thresholds of 8 hours, 16 hours, and 24 hours of wear-time.	

^a^N/A: not applicable.

### Strengths and Limitations

To our knowledge, this is the first systematic review to focus specifically on BioMeT adherence. Further strengths of our study include the large number of manuscripts screened for potential inclusion, and the quality control processes that we implemented, which resulted in few disagreements among the reviewers. The sensitive, rather than specific, search terms we adopted increased our confidence that we were able to capture the relevant set of literature, given our initial concern that many BioMeTs were used for exploratory analyses and therefore not referred to in study titles, abstracts, or keywords. The quality control process was particularly important, given that 76.4% (718/940) of the manuscripts were screened by a single investigator. Alongside these strengths, this review has several limitations that should be noted. Owing to the inconsistencies in the study outcome measures and variability in the definition of adherence adopted across studies, we did not undertake a methodological assessment and could not determine statistical inference. We also did not extract every element of the study design, such as the duration of the study itself or the length of time the BioMeTs were used per protocol. We included only peer reviewed publications indexed in PubMed; therefore, our findings may not be representative of all studies capturing BioMeT data in related fields, such as engineering. Finally, due to the vast number of manuscripts captured in our PubMed search terms, we limited the time frame to a 5-year period, and we limited our review to studies reporting nonsurrogate measurements of adherence, thereby excluding technology such as smart pill bottles which may offer valuable adherence data where a nonsurrogate measurement is not feasible.

### Conclusions

This review provides a description of the numerous methods that have been used in recent years to measure BioMeT adherence, allowing us to identify gaps and make specific reporting recommendations. Several important questions remain, which we hope will be addressed in future studies. For example, it will be interesting to compare our findings to a similar review covering the subsequent 5-year period (2020-2025), as the abrupt acceleration of digital monitoring and interventions including telemedicine during the current COVID-19 era [31,32] will likely, in hindsight, be considered a paradigm shift in both research and health care delivery. We hope that with increased consistency and reporting of data elements, it will become possible to meta-analyze adherence data to identify the possible determinants of BioMeT use patterns. Only when adherence data are adequately reported will the field of digital medicine be able to advance our understanding of the reasons underlying acceptance and adherence, which will ultimately allow investigators to optimize the design of tools, studies, implementation methods, and user engagement strategies to realize the full potential of BioMeTs as digital monitoring, diagnostic, and therapeutic tools. To support these actions, we recommend that (1) quantitative, nonsurrogate, sensor-based adherence data be reported for all BioMeTs when feasible; (2) a clear description of the sensor or sensors used to capture adherence data, the algorithm or algorithms that convert sample-level measurements to a metric of adherence, and the analytic validation data demonstrating that BioMeT-generated adherence is an accurate and reliable measurement of actual use be provided when available; and (3) primary adherence data be reported as a continuous variable followed by categorical definitions if needed, and that the categories adopted are supported by clinical validation data and/or consistent with previous reports.

## Appendix

Multimedia Appendix 1 PubMed search terms.

Multimedia Appendix 2 All manuscripts identified for data extraction.

## Abbreviations

BioMeT: biometric monitoring technology
DiMe: Digital Medicine Society
PICOS: Population, Intervention, Comparison, Outcomes, and Study design
