# Supplementary material for: Recommendations for Defining and Reporting Adherence Measured by Biometric Monitoring Technologies: Systematic Review
Source: J Med Internet Res. 2022 Apr 14;24(4):e33537. doi: 10.2196/33537 (PMC9052021; doi:10.2196/33537)
Supplement: Multimedia Appendix 1 [file jmir_v24i4e33537_app1.docx]

**Multimedia Appendix 1:** PubMed search terms

| Layer | Purpose | Search terms |
| --- | --- | --- |
| A | Used a BioMeT | ((((((("Diagnosis"[mesh] OR "Investigative Techniques"[mesh] OR "Equipment and supplies"[mesh] AND (wear*[tiab] OR worn*[tiab] OR wore[tiab] OR implantable[tiab] OR implanted[tiab] OR ingest*[tiab] OR portable[tiab] OR embedded[tiab] OR proxim*[tiab] OR wrist[tiab] OR cuff[tiab] OR patch[tiab] OR headband[tiab] OR watch[tiab] OR tracker[tiab] OR smartphone[tiab] OR phone[tiab] OR app[tiab] OR fitness[tiab] OR wellness[tiab] OR connected[tiab] OR Bluetooth[tiab] OR cloud[tiab] OR dock[tiab] OR mobile[tiab] OR wireless[tiab] OR wifi[tiab] OR digital[tiab] OR instrument[tiab] OR device[tiab] OR transduc*[tiab] OR sensor[tiab] OR biosensor[tiab] OR algorithm[tiab])))) |
| B | Reported adherence | AND ("Patient Compliance"[MeSH])) |
| C | Clinical studies | AND (("Clinical Study"[Publication Type]) OR ("Evaluation Studies"[Publication Type]) OR ("Comparative Study"[Publication Type]) OR ("Multicenter Study"[Publication Type]) OR ("Validation Study"[Publication Type]))) |
| D | Original data | NOT ((Editorial[Publication Type] OR Letter[Publication Type] OR Case Reports[Publication Type] OR Comment[Publication Type])) |
| E | Publication dates | AND ("2014/01/01"[Date - Publication] : "2019/10/31"[Date – Publication]))) AND ("2014/01/01"[mhda] : "2019/11/19"[mhda]) |
| Complete search:  ((((((("Diagnosis"[mesh] OR "Investigative Techniques"[mesh] OR "Equipment and supplies"[mesh] AND (wear*[tiab] OR worn*[tiab] OR wore[tiab] OR implantable[tiab] OR implanted[tiab] OR ingest*[tiab] OR portable[tiab] OR embedded[tiab] OR proxim*[tiab] OR wrist[tiab] OR cuff[tiab] OR patch[tiab] OR headband[tiab] OR watch[tiab] OR tracker[tiab] OR smartphone[tiab] OR phone[tiab] OR app[tiab] OR fitness[tiab] OR wellness[tiab] OR connected[tiab] OR Bluetooth[tiab] OR cloud[tiab] OR dock[tiab] OR mobile[tiab] OR wireless[tiab] OR wifi[tiab] OR digital[tiab] OR instrument[tiab] OR device[tiab] OR transduc*[tiab] OR sensor[tiab] OR biosensor[tiab] OR algorithm[tiab])))) AND ("Patient Compliance"[MeSH])) AND (("Clinical Study"[Publication Type]) OR ("Evaluation Studies"[Publication Type]) OR ("Comparative Study"[Publication Type]) OR ("Multicenter Study"[Publication Type]) OR ("Validation Study"[Publication Type]))) NOT ((Editorial[Publication Type] OR Letter[Publication Type] OR Case Reports[Publication Type] OR Comment[Publication Type])) AND ("2014/01/01"[Date - Publication] : "2019/10/31"[Date – Publication]))) AND ("2014/01/01"[mhda] : "2019/11/19"[mhda]) | | |
