# Supplementary material for: Recommendations for Defining and Reporting Adherence Measured by Biometric Monitoring Technologies: Systematic Review
Source: J Med Internet Res. 2022 Apr 14;24(4):e33537. doi: 10.2196/33537 (PMC9052021; doi:10.2196/33537)
Supplement: Multimedia Appendix 2 [file jmir_v24i4e33537_app2.docx]

**Multimedia Appendix 2:** All manuscripts identified for data extraction

| AUTHORS | TITLE | | REFERENCE | | PUBMED ID |
| --- | --- | --- | --- | --- | --- |
| Al Khatib HK, Hall WL, Creedon A, Ooi E, Masri T, McGowan L, Harding SV, Darzi J, Pot GK. | Sleep extension is a feasible lifestyle intervention in free-living adults who are habitually short sleepers: a potential strategy for decreasing intake of free sugars? A randomized controlled pilot study. | | Am J Clin Nutr. 2018 Jan 1;107(1):43-53. doi: 10.1093/ajcn/nqx030. Erratum in: Am J Clin Nutr. 2018 Apr 1;107(4):676. | | 29381788 |
| Amorim AB, Pappas E, Simic M, Ferreira ML, Jennings M, Tiedemann A, Carvalho-E-Silva AP, Caputo E, Kongsted A, Ferreira PH. | Integrating Mobile-health, health coaching, and physical activity to reduce the burden of chronic low back pain trial (IMPACT): a pilot randomised controlled trial. | | BMC Musculoskelet Disord. 2019 Feb 11;20(1):71. doi: 10.1186/s12891-019-2454-y. | | 30744606 |
| Anttalainen U, Melkko S, Hakko S, Laitinen T, Saaresranta T. | Telemonitoring of CPAP therapy may save nursing time. | | Sleep Breath. 2016 Dec;20(4):1209-1215. doi: 10.1007/s11325-016-1337-9. Epub 2016 Apr 4. | | 27043327 |
| Bakker JP, Wang R, Weng J, Aloia MS, Toth C, Morrical MG, Gleason KJ, Rueschman M, Dorsey C, Patel SR, Ware JH, Mittleman MA, Redline S. | Motivational Enhancement for Increasing Adherence to CPAP: A Randomized Controlled Trial. | | Chest. 2016 Aug;150(2):337-45. doi: 10.1016/j.chest.2016.03.019. Epub 2016 Mar 24. | | 27018174 |
| Bello C, Roseth A, Guardiola J, Reenaers C, Ruiz-Cerulla A, Van Kemseke C, Arajol C, Reinhard C, Seidel L, Louis E. | Usability of a home-based test for the measurement of fecal calprotectin in asymptomatic IBD patients. | | Dig Liver Dis. 2017 Sep;49(9):991-996. doi: 10.1016/j.dld.2017.05.009. Epub 2017 May 19. | | 28587751 |
| Berli C, Stadler G, Shrout PE, Bolger N, Scholz U. | Mediators of Physical Activity Adherence: Results from an Action Control Intervention in Couples. | | Ann Behav Med. 2018 Jan 5;52(1):65-76. doi: 10.1007/s12160-017-9923-z. | | 28710666 |
| Bros JS, Poulet C, Arnol N, Deschaux C, Gandit M, Charavel M. | Acceptance of Telemonitoring Among Patients with Obstructive Sleep Apnea Syndrome: How is the Perceived Interest by and for Patients? | | Telemed J E Health. 2018 May;24(5):351-359. doi: 10.1089/tmj.2017.0134. Epub 2017 Oct 13. | | 29027869 |
| Chai PR, Carreiro S, Innes BJ, Rosen RK, O'Cleirigh C, Mayer KH, Boyer EW. | Digital Pills to Measure Opioid Ingestion Patterns in Emergency Department Patients With Acute Fracture Pain: A Pilot Study. | | J Med Internet Res. 2017 Jan 13;19(1):e19. doi: 10.2196/jmir.7050. | | 28087496 |
| Chandler J, Sox L, Kellam K, Feder L, Nemeth L, Treiber F. | Impact of a Culturally Tailored mHealth Medication Regimen Self-Management Program upon Blood Pressure among Hypertensive Hispanic Adults. | | Int J Environ Res Public Health. 2019 Apr 6;16(7). pii: E1226. doi: 10.3390/ijerph16071226. | | 30959858 |
| Chen JH, Lauderdale DS. | Cognitive Function, Consent for Participation, and Compliance With Wearable Device Protocols in Older Adults. | | J Gerontol A Biol Sci Med Sci. 2019 Jan 16;74(2):269-273. doi: 10.1093/gerona/gly032. | | 29579176 |
| Ciemins EL, Arora A, Coombs NC, Holloway B, Mullette EJ, Garland R, Walsh Bishop-Green S, Penso J, Coon PJ. | Improving Blood Pressure Control Using Smart Technology. | | Telemed J E Health. 2018 Mar;24(3):222-228. doi: 10.1089/tmj.2017.0028. Epub 2017 Sep 30. | | 28930497 |
| Coker-Bolt P, Downey RJ, Connolly J, Hoover R, Shelton D, Seo NJ. | Exploring the feasibility and use of accelerometers before, during, and after a camp-based CIMT program for children with cerebral palsy. | | J Pediatr Rehabil Med. 2017;10(1):27-36. doi: 10.3233/PRM-170408. | | 28339408 |
| Copur AS, Erik Everhart D, Zhang C, Chen Z, Shekhani H, Mathevosian S, Loveless J, Watson E, Kadri I, Wallace L, Simon E, Fulambarker AM. | Effect of personality traits on adherence with positive airway pressure therapy in obstructive sleep apnea patients. | | Sleep Breath. 2018 May;22(2):369-376. doi: 10.1007/s11325-017-1559-5. Epub 2017 Aug 30. | | 28856525 |
| Daimee UA, Vermilye K, Moss AJ, Goldenberg I, Klein HU, McNitt S, Zareba W, Kutyifa V. | Experience with the wearable cardioverter-defibrillator in older patients: Results from the Prospective Registry of Patients Using the Wearable Cardioverter-Defibrillator. | | Heart Rhythm. 2018 Sep;15(9):1379-1386. doi: 10.1016/j.hrthm.2018.04.014. Epub 2018 Apr 18. | | 29678779 |
| de Bock M, Cooper M, Retterath A, Nicholas J, Ly T, Jones T, Davis E. | Continuous Glucose Monitoring Adherence: Lessons From a Clinical Trial to Predict Outpatient Behavior. | | J Diabetes Sci Technol. 2016 May 3;10(3):627-32. doi: 10.1177/1932296816633484. Print 2016 May. | | 26908570 |
| Di Bartolo P, Nicolucci A, Cherubini V, Iafusco D, Scardapane M, Rossi MC. | Young patients with type 1 diabetes poorly controlled and poorly compliant with self-monitoring of blood glucose: can technology help? Results of the i-NewTrend randomized clinical trial. | | Acta Diabetol. 2017 Apr;54(4):393-402. doi: 10.1007/s00592-017-0963-4. Epub 2017 Jan 30. | | 28138788 |
| Di Fabio DR, Blomme CK, Smith KM, Welk GJ, Campbell CG. | Adherence to physical activity guidelines in mid-pregnancy does not reduce sedentary time: an observational study. | | Int J Behav Nutr Phys Act. 2015 Feb 24;12:27. doi: 10.1186/s12966-015-0191-7. | | 25879428 |
| Dieltjens M, Verbruggen AE, Braem MJ, Wouters K, Verbraecken JA, De Backer WA, Hamans E, Van de Heyning PH, Vanderveken OM. | Determinants of Objective Compliance During Oral Appliance Therapy in Patients With Sleep-Disordered Breathing: A Prospective Clinical Trial. | | JAMA Otolaryngol Head Neck Surg. 2015 Oct;141(10):894-900. | | 26402736 |
| Dougherty CM, Glenny RW, Burr RL, Flo GL, Kudenchuk PJ. | Prospective randomized trial of moderately strenuous aerobic exercise after an implantable cardioverter defibrillator. | | Circulation. 2015 May 26;131(21):1835-42. doi: 10.1161/CIRCULATIONAHA.114.014444. Epub 2015 Mar 19. | | 25792557 |
| Dougherty CM, Luttrell MN, Burr RL, Kim M, Haskell WL. | Adherence to an Aerobic Exercise Intervention after an Implantable Cardioverter Defibrillator (ICD). | | Pacing Clin Electrophysiol. 2016 Feb;39(2):128-39. doi: 10.1111/pace.12782. Epub 2015 Dec 3. | | 26548341 |
| Ehrmann D, Spengler M, Jahn M, Niebuhr D, Haak T, Kulzer B, Hermanns N. | Adherence Over Time: The Course of Adherence to Customized Diabetic Insoles as Objectively Assessed by a Temperature Sensor. | | J Diabetes Sci Technol. 2018 May;12(3):695-700. doi: 10.1177/1932296817747618. Epub 2017 Dec 27. | | 29281893 |
| Eijsvogel MM, Ubbink R, Dekker J, Oppersma E, de Jongh FH, van der Palen J, Brusse-Keizer MG. | Sleep position trainer versus tennis ball technique in positional obstructive sleep apnea syndrome. | | J Clin Sleep Med. 2015 Jan 15;11(2):139-47. doi: 10.5664/jcsm.4460. | | 25515276 |
| El-Solh AA, Homish GG, Ditursi G, Lazarus J, Rao N, Adamo D, Kufel T. | A Randomized Crossover Trial Evaluating Continuous Positive Airway Pressure Versus Mandibular Advancement Device on Health Outcomes in Veterans With Posttraumatic Stress Disorder. | | J Clin Sleep Med. 2017 Nov 15;13(11):1327-1335. doi: 10.5664/jcsm.6808. | | 29065960 |
| Erath JW, Vamos M, Sirat AS, Hohnloser SH. | The wearable cardioverter-defibrillator in a real-world clinical setting: experience in 102 consecutive patients. | | Clin Res Cardiol. 2017 Apr;106(4):300-306. doi: 10.1007/s00392-016-1054-1. Epub 2016 Nov 25. | | 27888304 |
| Evans J, Papadopoulos A, Silvers CT, Charness N, Boot WR, Schlachta-Fairchild L, Crump C, Martinez M, Ent CB. | Remote Health Monitoring for Older Adults and Those with Heart Failure: Adherence and System Usability. | | Telemed J E Health. 2016 Jun;22(6):480-8. doi: 10.1089/tmj.2015.0140. Epub 2015 Nov 5. | | 26540369 |
| Franke KJ, Domanski U, Schroeder M, Jansen V, Artmann F, Weber U, Ettler R, Nilius G. | Telemonitoring of home exercise cycle training in patients with COPD. | | Int J Chron Obstruct Pulmon Dis. 2016 Nov 11;11:2821-2829. eCollection 2016. | | 27956829 |
| Frasnelli M, Baty F, Niedermann J, Brutsche MH, Schoch OD. | Effect of telemetric monitoring in the first 30 days of continuous positive airway pressure adaptation for obstructive sleep apnoea syndrome - a controlled pilot study. | | J Telemed Telecare. 2016 Jun;22(4):209-14. doi: 10.1177/1357633X15598053. Epub 2015 Aug 6. | | 26253747 |
| Frias J, Virdi N, Raja P, Kim Y, Savage G, Osterberg L. | Effectiveness of Digital Medicines to Improve Clinical Outcomes in Patients with Uncontrolled Hypertension and Type 2 Diabetes: Prospective, Open-Label, Cluster-Randomized Pilot Clinical Trial. | | J Med Internet Res. 2017 Jul 11;19(7):e246. doi: 10.2196/jmir.7833. | | 28698169 |
| Gallegos-Carrillo K, GarcÃa-PeÃ±a C, SalmerÃ³n J, Salgado-de-Snyder N, Lobelo F. | Brief Counseling and Exercise Referral Scheme: A Pragmatic Trial in Mexico. | | Am J Prev Med. 2017 Feb;52(2):249-259. doi: 10.1016/j.amepre.2016.10.021. Epub 2016 Dec 6. | | 27939238 |
| Gardner AW, Parker DE, Montgomery PS, Blevins SM. | Step-monitored home exercise improves ambulation, vascular function, and inflammation in symptomatic patients with peripheral artery disease: a randomized controlled trial. | | J Am Heart Assoc. 2014 Sep 18;3(5):e001107. doi: 10.1161/JAHA.114.001107. | | 25237048 |
| Gerber CN, Kunz B, van Hedel HJ. | Preparing a neuropediatric upper limb exergame rehabilitation system for home-use: a feasibility study. | | J Neuroeng Rehabil. 2016 Mar 23;13:33. doi: 10.1186/s12984-016-0141-x. | | 27008504 |
| Goyder E, Hind D, Breckon J, Dimairo M, Minton J, Everson-Hock E, Read S, Copeland R, Crank H, Horspool K, Humphreys L, Hutchison A, Kesterton S, Latimer N, Scott E, Swaile P, Walters SJ, Wood R, Collins K, Cooper C. | A randomised controlled trial and cost-effectiveness evaluation of 'booster' interventions to sustain increases in physical activity in middle-aged adults in deprived urban neighbourhoods. | | Health Technol Assess. 2014 Feb;18(13):1-210. doi: 10.3310/hta18130. | | 24571932 |
| Graco M, Green SE, Tolson J, Stevens B, Barnes M, Rigoni A, Henderson S, Nicholls C, Berlowitz DJ. | Worth the effort? Weighing up the benefit and burden of continuous positive airway pressure therapy for the treatment of obstructive sleep apnoea in chronic tetraplegia. | | Spinal Cord. 2019 Mar;57(3):247-254. doi: 10.1038/s41393-018-0210-z. Epub 2018 Oct 24. | | 30356181 |
| Grossman JA, Arigo D, Bachman JL. | Meaningful weight loss in obese postmenopausal women: a pilot study of high-intensity interval training and wearable technology. | | Menopause. 2018 Apr;25(4):465-470. doi: 10.1097/GME.0000000000001013. | | 29088015 |
| Gulati A, Oscroft N, Chadwick R, Ali M, Smith I. | The impact of changing people with sleep apnea using CPAP less than 4 h per night to a Bi-level device. | | Respir Med. 2015 Jun;109(6):778-83. doi: 10.1016/j.rmed.2015.01.020. Epub 2015 Feb 19. | | 25933913 |
| Hartman JE, Boezen HM, Zuidema MJ, de Greef MH, Ten Hacken NH. | Physical activity recommendations in patients with chronic obstructive pulmonary disease. | | Respiration. 2014;88(2):92-100. doi: 10.1159/000360298. Epub 2014 May 14. | | 24851826 |
| Hedermann G, Vissing CR, Heje K, Preisler N, Witting N, Vissing J. | Aerobic Training in Patients with Congenital Myopathy. | | PLoS One. 2016 Jan 11;11(1):e0146036. doi: 10.1371/journal.pone.0146036. eCollection 2016. | | 26751952 |
| Heiser C, Knopf A, Bas M, Gahleitner C, Hofauer B. | Selective upper airway stimulation for obstructive sleep apnea: a single center clinical experience. | | Eur Arch Otorhinolaryngol. 2017 Mar;274(3):1727-1734. doi: 10.1007/s00405-016-4297-6. Epub 2016 Sep 12. | | 27619823 |
| Heiser C, Maurer JT, Hofauer B, Sommer JU, Seitz A, Steffen A. | Outcomes of Upper Airway Stimulation for Obstructive Sleep Apnea in a Multicenter German Postmarket Study. | | Otolaryngol Head Neck Surg. 2017 Feb;156(2):378-384. doi: 10.1177/0194599816683378. Epub 2016 Dec 27. | | 28025918 |
| Hostler JM, Sheikh KL, Andrada TF, Khramtsov A, Holley PR, Holley AB. | A mobile, web-based system can improve positive airway pressure adherence. | | J Sleep Res. 2017 Apr;26(2):139-146. doi: 10.1111/jsr.12476. Epub 2016 Dec 8. | | 27933667 |
| Howie EK, Campbell AC, Straker LM. | An active video game intervention does not improve physical activity and sedentary time of children at-risk for developmental coordination disorder: a crossover randomized trial. | | Child Care Health Dev. 2016 Mar;42(2):253-60. doi: 10.1111/cch.12305. Epub 2015 Dec 9. | | 26648488 |
| Howie EK, McVeigh JA, Straker LM. | Comparison of Compliance and Intervention Outcomes Between Hip- and Wrist-Worn Accelerometers During a Randomized Crossover Trial of an Active Video Games Intervention in Children. | | J Phys Act Health. 2016 Sep;13(9):964-9. doi: 10.1123/jpah.2015-0470. Epub 2016 Aug 16. | | 27172616 |
| Huang K, Sparto PJ, Kiesler S, Siewiorek DP, Smailagic A. | iPod-based in-home system for monitoring gaze-stabilization exercise compliance of individuals with vestibular hypofunction. | | J Neuroeng Rehabil. 2014 Apr 21;11:69. doi: 10.1186/1743-0003-11-69. | | 24746068 |
| Huang YC, Lin CY, Lan CC, Wu YK, Lim CS, Huang CY, Huang HL, Yeh KH, Liu YC, Yang MC. | Comparison of cardiovascular co-morbidities and CPAP use in patients with positional and non-positional mild obstructive sleep apnea. | | BMC Pulm Med. 2014 Sep 26;14:153. doi: 10.1186/1471-2466-14-153. | | 25257571 |
| Hyun P, Preston CB, Al-Jewair TS, Park-Hyun E, Tabbaa S. | Patient compliance with Hawley retainers fitted with the SMART(Â®) sensor: a prospective clinical pilot study. | | Angle Orthod. 2015 Mar;85(2):263-9. doi: 10.2319/030814-163.1. Epub 2014 May 28. | | 24869901 |
| Jung ME, Bourne JE, Beauchamp MR, Robinson E, Little JP. | High-intensity interval training as an efficacious alternative to moderate-intensity continuous training for adults with prediabetes. | | J Diabetes Res. 2015;2015:191595. doi: 10.1155/2015/191595. Epub 2015 Mar 30. | | 25918728 |
| Karol LA, Virostek D, Felton K, Wheeler L. | Effect of Compliance Counseling on Brace Use and Success in Patients with Adolescent Idiopathic Scoliosis. | | J Bone Joint Surg Am. 2016 Jan 6;98(1):9-14. doi: 10.2106/JBJS.O.00359. | | 26738898 |
| Keukenkamp R, Merkx MJ, Busch-Westbroek TE, Bus SA. | An Explorative Study on the Efficacy and Feasibility of the Use of Motivational Interviewing to Improve Footwear Adherence in Persons with Diabetes at High Risk for Foot Ulceration. | | J Am Podiatr Med Assoc. 2018 Mar;108(2):90-99. doi: 10.7547/16-171. Epub 2017 Nov 7. | | 29111785 |
| Keytsman C, Van Noten P, Spaas J, Nieste I, Van Asch P, Eijnde BO. | Periodized home-based training: A new strategy to improve high intensity exercise therapy adherence in mildly affected patients with Multiple Sclerosis. | | Mult Scler Relat Disord. 2019 Feb;28:91-97. doi: 10.1016/j.msard.2018.12.018. Epub 2018 Dec 14. | | 30576848 |
| Kim JY, Wineinger NE, Steinhubl SR. | The Influence of Wireless Self-Monitoring Program on the Relationship Between Patient Activation and Health Behaviors, Medication Adherence, and Blood Pressure Levels in Hypertensive Patients: A Substudy of a Randomized Controlled Trial. | | J Med Internet Res. 2016 Jun 22;18(6):e116. doi: 10.2196/jmir.5429. | | 27334418 |
| Kraal JJ, Van den Akker-Van Marle ME, Abu-Hanna A, Stut W, Peek N, Kemps HM. | Clinical and cost-effectiveness of home-based cardiac rehabilitation compared to conventional, centre-based cardiac rehabilitation: Results of the FIT@Home study. | | Eur J Prev Cardiol. 2017 Aug;24(12):1260-1273. doi: 10.1177/2047487317710803. Epub 2017 May 23. | | 28534417 |
| Kuna ST, Shuttleworth D, Chi L, Schutte-Rodin S, Friedman E, Guo H, Dhand S, Yang L, Zhu J, Bellamy SL, Volpp KG, Asch DA. | Web-Based Access to Positive Airway Pressure Usage with or without an Initial Financial Incentive Improves Treatment Use in Patients with Obstructive Sleep Apnea. | | Sleep. 2015 Aug 1;38(8):1229-36. doi: 10.5665/sleep.4898. | | 25581921 |
| Lakshminarayan K, Westberg S, Northuis C, Fuller CC, Ikramuddin F, Ezzeddine M, Scherber J, Speedie S. | A mHealth-based care model for improving hypertension control in stroke survivors: Pilot RCT. | | Contemp Clin Trials. 2018 Jul;70:24-34. doi: 10.1016/j.cct.2018.05.005. Epub 2018 May 12. | | 29763657 |
| Landau Z, Abiri S, Gruber N, Levy-Shraga Y, Brener A, Lebenthal Y, Barash G, Pinhas-Hamiel O, Rachmiel M. | Use of flash glucose-sensing technology (FreeStyle Libre) in youth with type 1 diabetes: AWeSoMe study group real-life observational experience. | | Acta Diabetol. 2018 Dec;55(12):1303-1310. doi: 10.1007/s00592-018-1218-8. Epub 2018 Aug 31. Erratum in: Acta Diabetol. 2018 Sep 12;:. | | 30171412 |
| Langer D, Charususin N, JÃ¡come C, Hoffman M, McConnell A, Decramer M, Gosselink R. | Efficacy of a Novel Method for Inspiratory Muscle Training in People With Chronic Obstructive Pulmonary Disease. | | Phys Ther. 2015 Sep;95(9):1264-73. doi: 10.2522/ptj.20140245. Epub 2015 Apr 9. | | 25858974 |
| Laub RR, TÃ¸nnesen P, Jennum PJ. | A Sleep Position Trainer for positional sleep apnea: a randomized, controlled trial. | | J Sleep Res. 2017 Oct;26(5):641-650. doi: 10.1111/jsr.12530. Epub 2017 Mar 31. | | 28370716 |
| Laub RR, TÃ¸nnesen P, Jennum PJ. | | Better together: reduced compliance after sequential versus simultaneous bilateral hearing aids fitting. | | J Sleep Res. 2017 Oct;26(5):641-650. doi: 10.1111/jsr.12530. Epub 2017 Mar 31. | |
| Lauret GJ, Fokkenrood HJ, Bendermacher BL, Scheltinga MR, Teijink JA. | Physical activity monitoring in patients with intermittent claudication. | | Eur J Vasc Endovasc Surg. 2014 Jun;47(6):656-63. doi: 10.1016/j.ejvs.2014.03.001. Epub 2014 Apr 13. | | 24735778 |
| Leemans J, Rodenstein D, Bousata J, Mwenge GB. | Impact of purchasing the CPAP device on acceptance and long-term adherence: a Belgian model. | | Acta Clin Belg. 2018 Feb;73(1):34-39. doi: 10.1080/17843286.2017.1336294. Epub 2017 Jun 11. | | 28602146 |
| Leger D, Elbaz M, Piednoir B, Carron A, Texereau J. | Evaluation of the add-on NOWAPIÂ® medical device for remote monitoring of compliance to Continuous Positive Airway Pressure and treatment efficacy in obstructive sleep apnea. | | Biomed Eng Online. 2016 Feb 27;15:26. doi: 10.1186/s12938-016-0139-4. | | 26922498 |
| Leroux C, Gingras V, Desjardins K, Brazeau AS, Ott-Braschi S, Strychar I, Rabasa-Lhoret R. | In adult patients with type 1 diabetes healthy lifestyle associates with a better cardiometabolic profile. | | Nutr Metab Cardiovasc Dis. 2015 May;25(5):444-51. doi: 10.1016/j.numecd.2015.01.004. Epub 2015 Jan 28. | | 25770760 |
| Ling J, King KM. | Measuring Physical Activity of Elementary School Children With Unsealed Pedometers: Compliance, Reliability, and Reactivity. | | J Nurs Meas. 2015;23(2):271-86. doi: 10.1891/1061-3749.23.2.271. | | 26284840 |
| Lipsmeier F, Taylor KI, Kilchenmann T, Wolf D, Scotland A, Schjodt-Eriksen J, Cheng WY, Fernandez-Garcia I, Siebourg-Polster J, Jin L, Soto J, Verselis L, Boess F, Koller M, Grundman M, Monsch AU, Postuma RB, Ghosh A, Kremer T, Czech C, Gossens C, Lindemann M. | Evaluation of smartphone-based testing to generate exploratory outcome measures in a phase 1 Parkinson's disease clinical trial. | | Mov Disord. 2018 Aug;33(8):1287-1297. doi: 10.1002/mds.27376. Epub 2018 Apr 27. | | 29701258 |
| Locke SR, Bourne JE, Beauchamp MR, Little JP, Barry J, Singer J, Jung ME. | High-Intensity Interval or Continuous Moderate Exercise: A 24-Week Pilot Trial. | | Med Sci Sports Exerc. 2018 Oct;50(10):2067-2075. doi: 10.1249/MSS.0000000000001668. | | 29762252 |
| Lowres N, Mulcahy G, Gallagher R, Ben Freedman S, Marshman D, Kirkness A, Orchard J, Neubeck L. | Self-monitoring for atrial fibrillation recurrence in the discharge period post-cardiac surgery using an iPhone electrocardiogram. | | Eur J Cardiothorac Surg. 2016 Jul;50(1):44-51. doi: 10.1093/ejcts/ezv486. Epub 2016 Feb 4. | | 26850266 |
| Lutsey PL, Chen LY, Eaton A, Jaeb M, Rudser KD, Neaton JD, Alonso A. | A Pilot Randomized Trial of Oral Magnesium Supplementation on Supraventricular Arrhythmias. | | Nutrients. 2018 Jul 10;10(7). pii: E884. doi: 10.3390/nu10070884. | | 29996476 |
| Machaalani R, Evans CA, Waters KA. | Objective adherence to positive airway pressure therapy in an Australian paediatric cohort. | | Sleep Breath. 2016 Dec;20(4):1327-1336. doi: 10.1007/s11325-016-1400-6. Epub 2016 Sep 3. | | 27591801 |
| Malhotra A, Crocker ME, Willes L, Kelly C, Lynch S, Benjafield AV. | Patient Engagement Using New Technology to Improve Adherence to Positive Airway Pressure Therapy: A Retrospective Analysis. | | Chest. 2018 Apr;153(4):843-850. doi: 10.1016/j.chest.2017.11.005. Epub 2017 Nov 15. | | 29154970 |
| McDermott MM, Spring B, Berger JS, Treat-Jacobson D, Conte MS, Creager MA, Criqui MH, Ferrucci L, Gornik HL, Guralnik JM, Hahn EA, Henke P, Kibbe MR, Kohlman-Trighoff D, Li L, Lloyd-Jones D, McCarthy W, Polonsky TS, Skelly C, Tian L, Zhao L, Zhang D, et al. | Effect of a Home-Based Exercise Intervention of Wearable Technology and Telephone Coaching on Walking Performance in Peripheral Artery Disease: The HONOR Randomized Clinical Trial. | | JAMA. 2018 Apr 24;319(16):1665-1676. doi: 10.1001/jama.2018.3275. Erratum in: JAMA. 2018 Jul 3;320(1):96. | | 29710165 |
| Michaliszyn SF, Higgins M, Faulkner MS. | Patterns of Physical Activity Adherence by Adolescents With Diabetes or Obesity Enrolled in a Personalized Community-Based Intervention. | | Diabetes Educ. 2018 Dec;44(6):519-530. doi: 10.1177/0145721718805693. Epub 2018 Oct 11. | | 30306834 |
| Mitchell KE, Johnson V, Houchen-Wolloff L, Sewell L, Morgan MD, Steiner MC, Singh SJ. | Agreement between adherences to four physical activity recommendations in patients with COPD: does the incremental shuttle walk test predict adherence? | | Clin Respir J. 2018 Feb;12(2):510-516. doi: 10.1111/crj.12555. Epub 2016 Oct 13. | | 27717153 |
| Morgenstein A, Davis R, Talwalkar V, Iwinski H Jr, Walker J, Milbrandt TA. | A randomized clinical trial comparing reported and measured wear rates in clubfoot bracing using a novel pressure sensor. | | J Pediatr Orthop. 2015 Mar;35(2):185-91. doi: 10.1097/BPO.0000000000000205. | | 24787312 |
| Munafo D, Hevener W, Crocker M, Willes L, Sridasome S, Muhsin M. | A telehealth program for CPAP adherence reduces labor and yields similar adherence and efficacy when compared to standard of care. | | Sleep Breath. 2016 May;20(2):777-85. doi: 10.1007/s11325-015-1298-4. Epub 2016 Jan 11. | | 26754933 |
| Najafi B, Ron E, Enriquez A, Marin I, Razjouyan J, Armstrong DG. | Smarter Sole Survival: Will Neuropathic Patients at High Risk for Ulceration Use a Smart Insole-Based Foot Protection System? | | J Diabetes Sci Technol. 2017 Jul;11(4):702-713. doi: 10.1177/1932296816689105. Epub 2017 Jan 30. | | 28627227 |
| Nicolson PJA, Hinman RS, Wrigley TV, Stratford PW, Bennell KL. | Self-reported Home Exercise Adherence: A Validity and Reliability Study Using Concealed Accelerometers. | | J Orthop Sports Phys Ther. 2018 Dec;48(12):943-950. doi: 10.2519/jospt.2018.8275. Epub 2018 Jul 27. | | 30053792 |
| Nyrop KA, Deal AM, Choi SK, Wagoner CW, Lee JT, Wood WA, Anders C, Carey LA, Dees EC, Jolly TA, Reeder-Hayes KE, Muss HB. | Measuring and understanding adherence in a home-based exercise intervention during chemotherapy for early breast cancer. | | Breast Cancer Res Treat. 2018 Feb;168(1):43-55. doi: 10.1007/s10549-017-4565-1. Epub 2017 Nov 9. Erratum in: Breast Cancer Res Treat. 2019 Jan;173(1):245. | | 29124455 |
| Picard S, Hanaire H, Baillot-Rudoni S, Gilbert-Bonnemaison E, Not D, Reznik Y, Guerci B. | Evaluation of the Adherence to Continuous Glucose Monitoring in the Management of Type 1 Diabetes Patients on Sensor-Augmented Pump Therapy: The SENLOCOR Study. | | Diabetes Technol Ther. 2016 Mar;18(3):127-35. doi: 10.1089/dia.2015.0240. Epub 2016 Mar 7. | | 26950530 |
| Price L, Wyatt K, Lloyd J, Abraham C, Creanor S, Dean S, Hillsdon M. | Children's Compliance With Wrist-Worn Accelerometry Within a Cluster-Randomized Controlled Trial: Findings From the Healthy Lifestyles Programme. | | Pediatr Exerc Sci. 2018 May 1;30(2):281-287. doi: 10.1123/pes.2017-0179. Epub 2018 Feb 8. | | 29417877 |
| Ramotowska A, Szypowska A. | Bolus calculator and wirelessly communicated blood glucose measurement effectively reduce hypoglycaemia in type 1 diabetic children - randomized controlled trial. | | Diabetes Metab Res Rev. 2014 Feb;30(2):146-53. doi: 10.1002/dmrr.2477. | | 24115314 |
| Ribeiro DC, Sole G, Abbott JH, Milosavljevic S. | The effectiveness of a lumbopelvic monitor and feedback device to change postural behavior: a feasibility randomized controlled trial. | | J Orthop Sports Phys Ther. 2014 Sep;44(9):702-11. doi: 10.2519/jospt.2014.5009. Epub 2014 Aug 6. | | 25098195 |
| Rowlands AV, Harrington DM, Bodicoat DH, Davies MJ, Sherar LB, Gorely T, Khunti K, Edwardson CL. | Compliance of Adolescent Girls to Repeated Deployments of Wrist-Worn Accelerometers. | | Med Sci Sports Exerc. 2018 Jul;50(7):1508-1517. doi: 10.1249/MSS.0000000000001588. | | 29474208 |
| Schott TC, Fritz U, Meyer-Gutknecht H. | Maxillary expansion therapy with plates featuring a transverse screw: implications of patient compliance with wear-time and screw activation requirements. | | J Orofac Orthop. 2014 Mar;75(2):107-17. doi: 10.1007/s00056-013-0197-1. Epub 2014 Mar 5. English, German. | | 24589750 |
| Schott TC, Ludwig B. | Microelectronic wear-time documentation of removable orthodontic devices detects heterogeneous wear behavior and individualizes treatment planning. | | Am J Orthod Dentofacial Orthop. 2014 Aug;146(2):155-60. doi: 10.1016/j.ajodo.2014.04.020. | | 25085297 |
| Sedkaoui K, Leseux L, Pontier S, Rossin N, Leophonte P, Fraysse JL, Didier A. | Efficiency of a phone coaching program on adherence to continuous positive airway pressure in sleep apnea hypopnea syndrome: a randomized trial. | | BMC Pulm Med. 2015 Sep 14;15:102. doi: 10.1186/s12890-015-0099-7. | | 26370444 |
| Sen AP, Sewell TB, Riley EB, Stearman B, Bellamy SL, Hu MF, Tao Y, Zhu J, Park JD, Loewenstein G, Asch DA, Volpp KG. | Financial incentives for home-based health monitoring: a randomized controlled trial. | | J Gen Intern Med. 2014 May;29(5):770-7. doi: 10.1007/s11606-014-2778-0. Epub 2014 Feb 13. | | 24522623 |
| Seneviratne SN, Jiang Y, Derraik J, McCowan L, Parry GK, Biggs JB, Craigie S, Gusso S, Peres G, Rodrigues RO, Ekeroma A, Cutfield WS, Hofman PL. | Effects of antenatal exercise in overweight and obese pregnant women on maternal and perinatal outcomes: a randomised controlled trial. | | BJOG. 2016 Mar;123(4):588-97. doi: 10.1111/1471-0528.13738. Epub 2015 Nov 6. | | 26542419 |
| Serra MC, Treuth MS, Ryan AS. | Dietary prescription adherence and non-structured physical activity following weight loss with and without aerobic exercise. | | J Nutr Health Aging. 2014 Dec;18(10):888-93. doi: 10.1007/s12603-014-0481-9. | | 25470804 |
| Sharma S, Mather P, Gupta A, Reeves G, Rubin S, Bonita R, Chowdhury A, Malloy R, Willes L, Whellan D. | Effect of Early Intervention With Positive Airway Pressure Therapy for Sleep Disordered Breathing on Six-Month Readmission Rates in Hospitalized Patients With Heart Failure. | | Am J Cardiol. 2016 Mar 15;117(6):940-5. doi: 10.1016/j.amjcard.2015.12.032. Epub 2015 Dec 31. | | 26830259 |
| Simmons ES, Paul R, Shic F. | Brief Report: A Mobile Application to Treat Prosodic Deficits in Autism Spectrum Disorder and Other Communication Impairments: A Pilot Study. | | J Autism Dev Disord. 2016 Jan;46(1):320-327. doi: 10.1007/s10803-015-2573-8. | | 26329637 |
| Smith YK, Verrett RG. | Evaluation of a novel device for measuring patient compliance with oral appliances in the treatment of obstructive sleep apnea. | | J Prosthodont. 2014 Jan;23(1):31-8. doi: 10.1111/jopr.12076. Epub 2013 Jul 25. | | 23889695 |
| Spring B, Pellegrini CA, Pfammatter A, Duncan JM, Pictor A, McFadden HG, Siddique J, Hedeker D. | Effects of an abbreviated obesity intervention supported by mobile technology: The ENGAGED randomized clinical trial. | | Obesity (Silver Spring). 2017 Jul;25(7):1191-1198. doi: 10.1002/oby.21842. Epub 2017 May 11. | | 28494136 |
| Takasaki H, Aoki S, May S. | No increase in 6-week treatment effect of Mechanical Diagnosis and Therapy with the use of the LUMOback in people with non-acute non-specific low back pain and a directional preference of extension: a pilot randomized controlled trial. | | Physiotherapy. 2018 Sep;104(3):347-353. doi: 10.1016/j.physio.2018.06.001. Epub 2018 Jun 8. | | 30031553 |
| Uhl JF, Benigni JP, Chahim M, FrÃ©deric D. | Prospective randomized controlled study of patient compliance in using a compression stocking: Importance of recommendations of the practitioner as a factor for better compliance. | | Phlebology. 2018 Feb;33(1):36-43. doi: 10.1177/0268355516682886. Epub 2016 Dec 9. | | 27940899 |
| van Zeller M, EusÃ©bio E, Almeida J, Winck JC. | Evaluation of adherence to ambulatory liquid oxygen treatment: are commercialized dual-pressure transducers helpful? | | Respir Care. 2014 Sep;59(9):1376-80. doi: 10.4187/respcare.02833. Epub 2014 Apr 29. | | 24782554 |
| Vella CA, Taylor K, Drummer D. | High-intensity interval and moderate-intensity continuous training elicit similar enjoyment and adherence levels in overweight and obese adults. | | Eur J Sport Sci. 2017 Oct;17(9):1203-1211. doi: 10.1080/17461391.2017.1359679. Epub 2017 Aug 9. | | 28792851 |
| Visser M, Brychta RJ, Chen KY, Koster A. | Self-reported adherence to the physical activity recommendation and determinants of misperception in older adults. | | J Aging Phys Act. 2014 Apr;22(2):226-34. doi: 10.1123/japa.2012-0219. Epub 2013 May 22. | | 23752449 |
| Wang JJ, Baranowski T, Lau PWC, Buday R, Gao Y. | Story Immersion May Be Effective in Promoting Diet and Physical Activity in Chinese Children. | | J Nutr Educ Behav. 2017 Apr;49(4):321-329.e1. doi: 10.1016/j.jneb.2017.01.001. | | 28391798 |
| Whitling S, Lyberg-Ã…hlander V, Rydell R. | Absolute or relative voice rest after phonosurgery: a blind randomized prospective clinical trial. | | Logoped Phoniatr Vocol. 2018 Dec;43(4):143-154. doi: 10.1080/14015439.2018.1504985. Epub 2018 Sep 5. | | 30183437 |
| Wilbur J, Miller AM, Fogg L, McDevitt J, Castro CM, Schoeny ME, Buchholz SW, Braun LT, Ingram DM, Volgman AS, Dancy BL. | Randomized Clinical Trial of the Women's Lifestyle Physical Activity Program for African-American Women: 24- and 48-Week Outcomes. | | Am J Health Promot. 2016 May;30(5):335-45. doi: 10.1177/0890117116646342. | | 27404642 |
| Wilbur J, Schoeny ME, Buchholz SW, Fogg L, Miller AM, Braun LT, Halloway S, Dancy BL. | Women's Lifestyle Physical Activity Program for African American Women: Fidelity Plan and Outcomes. | | J Phys Act Health. 2016 Oct;13(10):1100-1109. Epub 2016 Aug 16. | | 27256816 |
